# Metabolism of Skin-Absorbed Resveratrol into Its Glucuronized Form in Mouse Skin

**DOI:** 10.1371/journal.pone.0115359

**Published:** 2014-12-15

**Authors:** Itsuo Murakami, Romanas Chaleckis, Tomáš Pluskal, Ken Ito, Kousuke Hori, Masahiro Ebe, Mitsuhiro Yanagida, Hiroshi Kondoh

**Affiliations:** 1 Geriatric unit, Kyoto University Hospital, Sakyo-ku, Kyoto, Japan; 2 G0 Cell Unit, Okinawa Institute of Science and Technology Graduate University (OIST), Onna-son, Okinawa, Japan; Kinki University School of Pharmaceutical Sciences, Japan

## Abstract

Resveratrol (RESV) is a plant polyphenol, which is thought to have beneficial metabolic effects in laboratory animals as well as in humans. Following oral administration, RESV is immediately catabolized, resulting in low bioavailability. This study compared RESV metabolites and their tissue distribution after oral uptake and skin absorption. Metabolomic analysis of various mouse tissues revealed that RESV can be absorbed and metabolized through skin. We detected sulfated and glucuronidated RESV metabolites, as well as dihydroresveratrol. These metabolites are thought to have lower pharmacological activity than RESV. Similar quantities of most RESV metabolites were observed 4 h after oral or skin administration, except that glucuronidated RESV metabolites were more abundant in skin after topical RESV application than after oral administration. This result is consistent with our finding of glucuronidated RESV metabolites in cultured skin cells. RESV applied to mouse ears significantly suppressed inflammation in the TPA inflammation model. The skin absorption route could be a complementary, potent way to achieve therapeutic effects with RESV.

## Introduction

With increasing life expectancy [1], a concomitant increase of bed-ridden state patients is a rising social and clinical problem in Japan and other countries [2]. Major causes of the increase in bed-ridden patients are brain infarction, frailty, bone fracture, and others [3]. Many of these events are closely correlated with consequences of human ageing, such as atherosclerosis, osteoporosis, sarcopenia etc. [4], [5]. Prevention of such lifestyle-related diseases in the elderly is essential to avoid being bed-ridden. Information from basic research on ageing can help to mitigate this problem.

Various models of ageing have been proposed in the last century, including the radical theory of ageing [6], replicative senescence [7], and others [8]. One of the most widely accepted is a calorie restriction model. Since the initial discoveries by McCay and colleagues [9], calorie restriction is now well known to extend the lifespan of many species, including rats, fish, fruit flies, nematodes among others [10].

The calorie-restricted condition affects several key metabolites. Elevated respiration during calorie restriction is a mode of energy production that creates nicotinamide adenine dinucleotide (NAD) as a by-product and lowers levels of the reduced form, NADH [11]. NAD accumulation in a calorie-restricted condition activates NAD-dependent deacetylases, Sir2 and its mammalian homolog, Sirt1 [12], which are involved in age-related diseases [13]. Calorie restriction also turns on a gene called PNC1, which produces an enzyme that rids cells of nicotinamide, a small molecule similar to vitamin B3 that normally represses Sir2 [14]. Moreover, low-glucose or calorie-restricted conditions increase the AMP/ATP ratio, followed by activation of adenosine monophosphate-activated protein kinase (AMPK), a critical energy sensor that maintains cellular energy balance and regulates metabolic functions [15].

Thus the significance of calorie restriction in experimental longevity is well established, while its clinical application is more controversial. First, there is little evidence to suggest that calorie restriction could extend life spans of humans or primates [16]. Second, calorie restriction might improve some metabolic diseases (diabetes, obesity, etc), while it might lead to lower body mass index, which exacerbates sarcopenia, frailty, and other consequences of ageing [17]. Thus calorie restriction alone might not be a viable approach to increase human longevity or to prevent people from becoming bed-ridden. As an alternative, several substances are of interest to researchers to mimic the calorie-restricted state [18]. Among them, resveratrol (RESV) is a plant polyphenolic compound, present in grapes, berries and peanuts, that acts as an anti-oxidant and as an activator of SIRT1 and AMPK [19]. RESV improves cardiovascular health [20], [21], diabetes [22], inflammation, and cancer [23] and increases longevity in laboratory animals [24].

However, there have been few randomized clinical trials to examine health benefits of RESV and results have not been strongly conclusive [25],[26]. Moderate pharmacological effects of RESV in humans may result partly from its low bioavailability because of its extensive catabolism to sulfated, glucuronide-conjugated, or hydrogenated forms in liver and gut [27]–[31]. While most of studies of RESV treatment employed oral administration or gastric injection, few studies used alternative administration routes (e.g. skin absorption) [32]. Other routes should be considered, especially for bed-ridden patients, for several reasons. First, oral administration of any drug can be quite difficult due to dysphagia, a frequent symptom. Second, direct delivery via topical application might be more effective than oral treatment in preventing sarcopenia or frailty, which often cause patients to become bed-ridden. Moreover, topically applied RESV might be less affected by liver and gut metabolism. However, no detailed comparison of RESV metabolism and tissue distribution has been undertaken to compare oral administration and dermal absorption of RESV.

Here we report the comparison of RESV metabolites in mice administered orally and transdermally. We investigated RESV metabolites in cultured cells from various tissues. These data suggest overlapping, but distinct, tissue-dependent, metabolic profiles of RESV.

## Materials and Methods

### Chemicals and reagents

Reagents were obtained as follows: trans-resveratrol (RESV) for mouse and cell culture experiments (Tokyo Chemical Industry); standards for mass spectrometry, trans-resveratrol-3-O-sulfate (RESV-SULF), dihydroresveratrol (DH-RESV) (Santa Cruz Biotechnology); trans-resveratrol-3-O-glucuronide, (trans-RESV-3-O-GLUC), trans-resveratrol-4-O-glucuronide (trans-RESV-4-O-GLUC), cis–resveratrol-3-O-glucuronide (cis-RESV-3-O-GLUC) and cis-resveratrol-4-O-glucuronide (cis-RESV-4-O-GLUC) (Toronto Research Chemicals); Hydrophilic ointment (Nihonkampo). Hydrophilic ointment was prepared as described in Japanese pharmacopeia [33]. Carboxymethylcellulose (CMC) gel was prepared by mixing 20 g sodium carboxymethylcellulose salt, 100 mL glycerin, 380 mL water. Chemicals used in LC-MS analysis were HPLC or MS grade and were obtained from Wako Pure Chemical Industries.

### Animals and diets

Male 7–8-week HR-1 hairless mice (Japan SLC) and female 8-week C57BL/6 mice (Charles River Laboratories Japan) were kept on a 12 h light-dark cycle and given free access to food (F-2) and tap water. Animal experiments were approved by the Animal Research Committee, Graduate School of Medicine, Kyoto University, Japan.

### Mouse oral and skin resveratrol administration

RESV was dissolved in ethanol (50 mg/mL) and stored at −20°C. For oral administration, 1 mg RESV in ethanol was diluted in saline (total volume 200 µL). RESV was dosed about 40 mg/kg body weight, similar to the dosage used in human experiments [34]. For skin treatment, 1 mg RESV in ethanol was directly applied on the dorsal skin or mixed with 230 mg of base (0.4% RESV) and then swabbed onto the dorsal skin (around 2 cm^2^) without using anesthesia. Mice were separated from each other and could not lick RESV from their dorsal skin.

### Resveratrol treatment in cell culture for metabolomic analysis

HepG2 (human hepatoblastoma cells) [35], HaCaT (human keratinocytes) [36] and C2C12 (mouse myoblasts) [37] were used to examine RESV metabolism. Cells were maintained in Dulbecco’s Modified Eagle Medium (DMEM) with 1% penicillin/streptomycin and 2 mM L-glutamine with some modifications as follows; DMEM (1.0 g/L glucose) with 10% fetal calf serum (FCS) for HepG2; DMEM (4.5 g/L glucose) with 10% FCS for HaCaT; DMEM (4.5 g/L glucose) with 20% FCS for C2C12. C2C12 cells were differentiated into myotubes by switching medium into DMEM containing 2% horse serum. Cells were plated on a 10 cm dish and allowed to adhere for 18 h. Cells were treated with 20 or 200 µM RESV. After 4 h incubation, cells were washed with PBS twice, followed by addition of 2 mL 50% ice-cold methanol. Collected cells were transferred into tubes on ice and further processed as described in metabolome sample preparation section.

### Mouse tissue sample preparation for metabolomic analysis

Mice were anesthetized with pentobarbital and blood was collected from the right ventricle. For metabolite extraction 0.2 mL of blood were mixed with 1.8 mL 55% methanol, pre-chilled to −40°C and kept until further processing as described in the metabolome sample preparation section. In addition to blood, metabolome samples were prepared from liver, dorsal skin, leg skin, and hind limb muscle.

Around 100 mg of each mouse tissue was dissected and weighed. Tissues were minced and homogenized for 3 min in 500 µL 50% methanol on ice using a BioMasher II (Nippi, Tokyo, Japan). Next, 1 mL 50% methanol and internal standards (10 nmol 4-(2-hydroxyethyl)-1-piperazineethanesulfonic acid (HEPES) and piperazine-N,N’-bis(2-ethanesulfonic acid) (PIPES)) were added to the samples. HEPES and PIPES were used as internal standards to correct for the variation of sample preparation and different detection efficiency. After brief vortexing, samples were centrifuged at 500 g for 10 min at 4°C. Supernatant was recovered for further processing as described in the metabolome sample preparation section (without the repeated addition of internal standards and vortexing).

### Metabolome sample preparation

Metabolome sample preparation was previously described [38]. After addition of internal standards (10 nmol HEPES and PIPES) to metabolome samples and brief vortexing, samples were transferred to Amicon Ultra 10 kDa cut-off filters (Millipore, Billerica, MA, USA) to remove proteins. Following sample concentration by vacuum evaporation, each sample was resuspended in 40 µL 50% acetonitrile and 1 µL was used for liquid chromatography-mass spectrometry (LC-MS) analysis.

### LC-MS analysis

LC-MS data were obtained using a Paradigm MS4 HPLC system (Michrom Bioresources, Auburn, CA, USA) coupled to an LTQ Orbitrap mass spectrometer (Thermo Fisher Scientific, Waltham, MA, USA), as described previously [38]. Briefly, metabolites were separated on a ZIC-pHILIC column (Merck SeQuant, Sweden; 150×2.1 mm, 5 µm particle size) using acetonitrile (A) and 10 mM ammonium carbonate buffer, pH 9.3 (B) as the mobile phase, with gradient elution from 80% A to 20% A in 30 min, at a flow rate of 100 µL min^−1^. Peak areas of metabolites of interest were measured using the MZmine 2.10 software [39] and normalized using the peak areas of HEPES and PIPES, as described in S2 Table.

### Metabolite identification and data analysis

Peaks were assigned to metabolites based upon their retention times and *m/z* values. Compounds were confirmed with standards (STD) or with analysis of MS/MS spectra (MS/MS), if no standard was available. In cases where no standard compound or MS/MS spectrum was available, compounds were identified by generating chemical formulae using *m/z* values in MZmine 2.10 module [40] and given MS status. Data were analyzed using the scatter plot function built into MZmine 2.10 software [39], as well as by exporting the data to a spreadsheet.

### TPA-induced ear edema

The ear edema model was employed as previously described [41]. Briefly, 1 µg 12-O-Tetradecanoylphorbol-13-acetate (TPA) was applied to the right ear of female C57BL/6 mice. Edema was evaluated by measuring ear thickness and ear weight. Thirty minutes after TPA treatment, 1 mg RESV was applied to the inner and outer surfaces of the right ear, or was supplied orally. Ear thickness was measured with a micrometer before and 24 h after TPA treatment. After sacrificing mice with CO_2_, ear punch biopsies (6 mm diameter) were weighed.

### Statistical Analysis

Data are presented as means ± standard deviations (SD) of three or four samples. Statistical analysis was performed using an unpaired Student’s t-test, or one-way ANOVA followed by Dunnett’s test or Tukey’s test for comparison of multiple treatment groups. Statistical significance was established at P<0.05.

## Results

### Resveratrol metabolites detected after resveratrol treatments

In a series of mouse tissues and cell culture experiments we were able to detect RESV and various RESV metabolites from RESV-treated samples (Table 1, S1 Table, S1–S5 Figures). We confirmed the presence of RESV-SULF (307.027 *m/z*), a sulfate-conjugated RESV, in RESV-treated mice using a standard and MS/MS analysis (S1 Figure). Similarly, we confirmed the presence of dihydroresveratrol-sulfate (DH-RESV-SULF) by MS/MS analysis (S2 Figure). DH-RESV is a metabolite of RESV, formed by hydrogenation of the double bond of RESV by intestinal bacteria [28]. Retention times of peaks of free RESV and DH-RESV coincided with those of sulfated forms, so that quantification of free RESV and DH-RESV was obstructed in samples containing sulfated metabolites (S1 Figure, S2 Figure and S6 Figure). Due to this limitation we have not reported data on free RESV and DH-RESV, because most of the RESV-treated samples contained sulfated RESV metabolites.

**Table 1:** Detected RESV and DH-RESV metabolites were identified using standard compounds (STD), MS/MS spectrum analysis (MS/MS), or *m/z* value (MS).

Resveratrol metabolite	Abbreviation	Identification
Resveratrol-sulfate	RESV-SULF	STD (S5 Figure)
trans-Resveratrol-3-O- glucuronide	trans-RESV-3-O-GLUC	STD (S3 Figure)
cis-Resveratrol-3-O-glucuronide	cis-RESV-3-O-GLUC	STD (S3 Figure)
Resveratrol-sulfoglucuronide	RESV-SULF-GLUC	MS/MS (S4 Figure)
Resveratrol-disulfate	RESV-DISULF	MS (S4 Figure)
Resveratrol-diglucuronide	RESV-DIGLUC	MS (S4 Figure)
Dihydroresveratrol-sulfate	DH-RESV-SULF	MS/MS (S2 Figure)
Dihydroresveratrol- glucuronide	DH-RESV-GLUC	MS/MS (S5 Figure)
Dihydroresveratrol- sulfoglucuronide	DH-RESV-SULF-GLUC	MS (S5 Figure)

Two separate peaks corresponding to the mass (403.104 *m/z*) of RESV-glucuronide (glucuronic acid conjugated to the hydroxyl group of RESV) were detected in RESV-treated mice and tissue culture samples. After careful comparison with standards (S3 Figure), these could be identified as cis-RESV-3-O-glucuronide (cis-RESV-3-O-GLUC) and trans-RESV-3-O-glucuronide (trans-RESV-3-O-GLUC). cis-RESV-4-O-glucuronide and trans-RESV-4-O-glucuronide were barely detected in mouse and cell culture samples, as previously reported [42]. These results suggest that isomerization of RESV occurs in mouse or cultured cells either before or after glucuronidation.

In addition, using MS/MS or MS analysis we identified further metabolites of RESV (RESV-sulfoglucuronides, RESV-disulfate, RESV-diglucuronide) and DH-RESV (DH-RESV-glucuronide, DH-RESV-sulfoglucuronides) (S4–S5 Figures). Our mass spectrometry data for RESV and DH-RESV metabolites correspond to MS/MS data in previous studies (S1 Table).

In conclusion, RESV-SULF, trans-RESV-3-O-GLUC, cis-RESV-3-O-GLUC, DH-RESV-SULF and DH-RESV-GLUC peaks were clearly detected by our LC-MS method in RESV-treated mouse samples.

### Distribution of resveratrol and its metabolites in the mouse tissues

To analyze the distribution of RESV and its metabolites in mouse tissues, we administered 1 mg trans-RESV to mice via oral and dermal routes and collected tissues 4 h later for LC-MS analysis. In this experiment, we identified 132 distinct physiological metabolites (123 verified with standards) in mouse tissues (blood, liver, muscle, leg skin, dorsal skin) (data deposited in the MetaboLights repository, as described in the Supplemental materials section). Metabolites included nucleotides, amino acids, sugar-phosphates, vitamins, organic acids, etc. The majority of these identified metabolites (103/132 = 78%) were detectable in both negative and positive ionization modes. Significant metabolic changes after RESV-treatment both via oral and skin routes were not observed.

**Figure 1:**
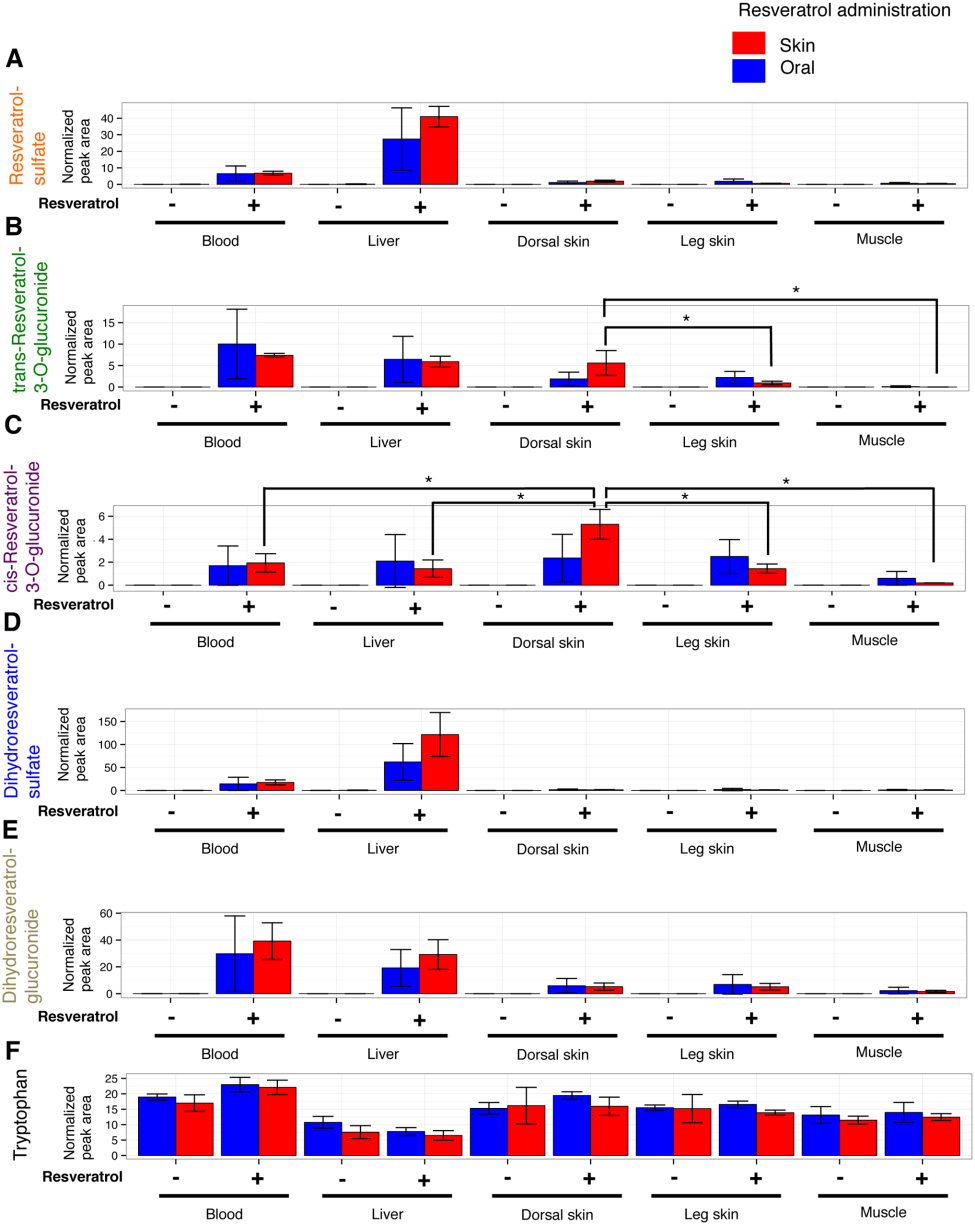
Distribution of RESV and its metabolite peaks in mouse tissues after oral and dermal administration. RESV (1 mg in 200 µL saline) was administered orally to hairless mice, or 1 mg RESV dissolved in ethanol was applied to the dorsal skin of hairless mice. After 4 h mice were sacrificed, metabolites were extracted from tissues and analyzed by LC-MS. Peak areas of RESV-SULF (**A**), trans-RESV-3-O-GLUC (**B**), cis-RESV-3-O-GLUC (**C**), DH-RESV-SULF (**D**) DH-RESV-GLUC (**E**), and tryptophan (**F**) were normalized by peak areas of spiked internal standards (HEPES and PIPES). Peak areas for each compound are presented as means ± standard deviation (SD) of 3 mice. Statistical significance was assessed by Student’s t-test or Tukey’s test: *P<0.05.

RESV-SULF and DH-RESV-SULF were detected mainly in liver and blood after both, oral- and skin-administration of RESV. RESV-SULF (Fig. 1A) and DH-RESV-SULF (Fig. 1D), were barely detectable in dorsal and leg skin, and muscle, regardless of RESV administration route. DH-RESV-GLUC (Fig. 1E) was also detected in blood and liver as well as at lower levels in skin and muscle after oral and skin administration of RESV. Thus RESV-SULF, DH-RESV-SULF and DH-RESV-GLUC showed comparable tissue distribution profiles regardless of administration route. These data indicate that RESV is absorbed orally and transdermally with similar efficiency and that its metabolites are detectable in a tissue-specific manner.

We observed strikingly distinct profiles for trans-RESV-3-O-GLUC and cis-RESV-3-O-GLUC depending upon administration route (Fig. 1B and 1C). After oral administration, trans-RESV-3-O-GLUC was detected mainly in blood and liver, however, after dermal application of RESV, we observed higher levels of trans-RESV-3-O-GLUC in dorsal skin, as well as in blood and liver. Interestingly, after dermal application of RESV, higher levels of cis-RESV-3-O-GLUC were detected in dorsal skin compared to other tissues. In contrast, after oral administration more or less equal levels of cis-RESV-3-O-GLUC were found in blood, liver, dorsal skin and leg skin. We noticed that in the case of transdermal administration, trans-RESV-3-O-GLUC and cis-RESV-3-O-GLUC were detected at higher levels in dorsal skin where RESV was applied, but at lower levels in leg skin, distant from dorsal skin. These data suggest that skin cells may be able to directly metabolize RESV into RESV glucuronides, which has not been reported previously.

### Resveratrol metabolism in cell culture

While the tissue distribution of most RESV metabolites after oral and skin administration of RESV is quite similar, distinct profiles of trans-RESV-3-O-GLUC and cis-RESV-3-O-GLUC in skin indicate that tissues other than liver and gut, (e.g. skin) may also be involved in RESV metabolism. To assess whether RESV metabolism is cell type-dependent, we analyzed RESV metabolism in liver, skin, and muscle cell cultures. We treated HepG2 (human hepatocyte), HaCaT (human keratinocyte) and C2C12 (mouse myoblast) cell lines with 20 or 200 µM trans-RESV for 4 h followed by metabolite extraction.

**Figure 2:**
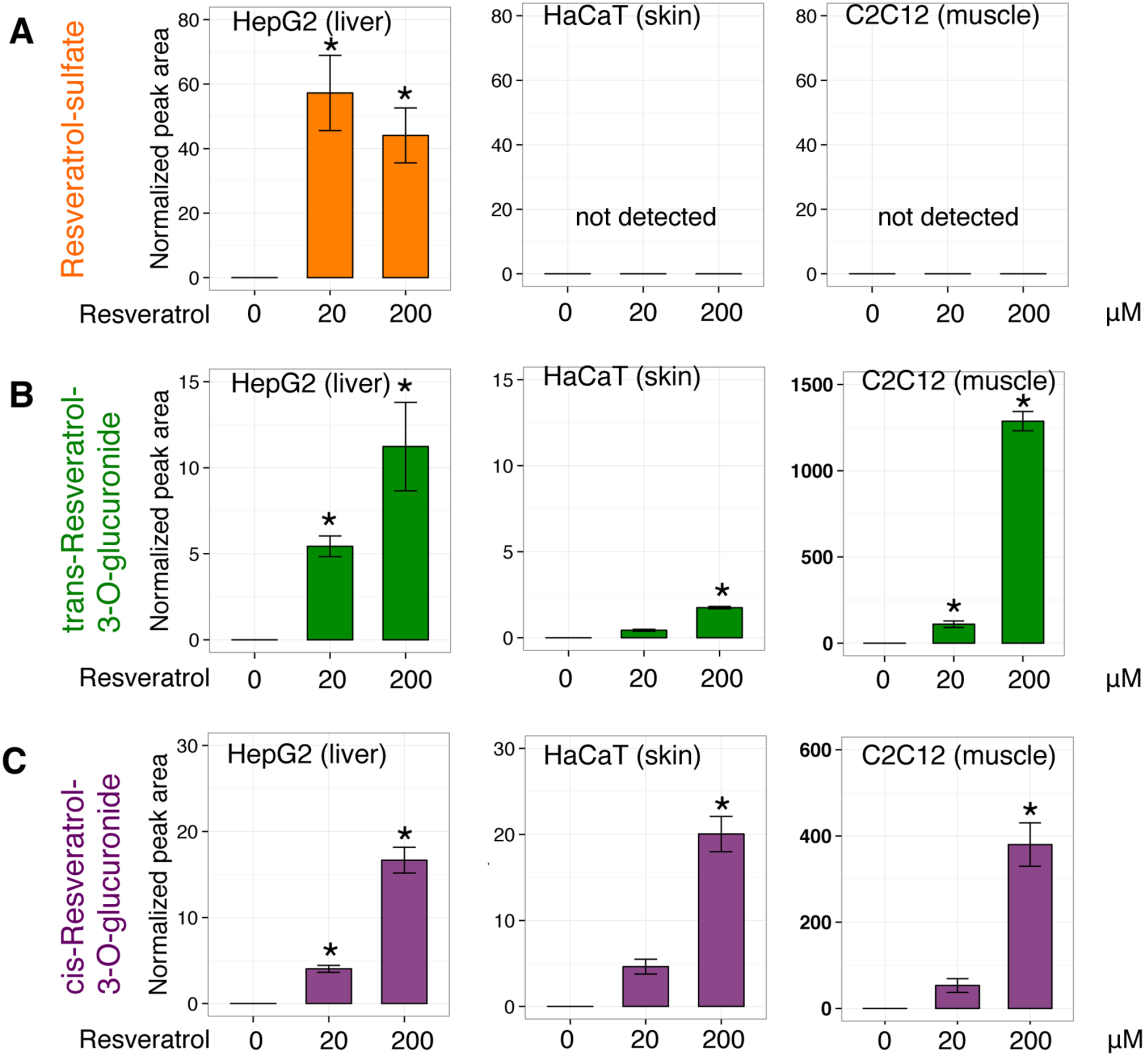
RESV metabolism in HepG2 (human hepatocytes), HaCaT (human keratinocytes), and C2C12 (mouse myoblasts). Cells were treated with 20 or 200 µM RESV for 4 h. After washing with PBS, cells were lysed, and metabolites were extracted and analyzed by LC-MS. Peak areas of RESV–SULF (**A**), trans-RESV-3-O-GLUC (**B**) and cis-RESV-3-O-GLUC (**C**) were normalized by peak areas of spiked internal standards (HEPES and PIPES). Peak areas for each compound are presented as means ± SD of 3 samples (except for HaCaT 200 µM RESV, 2 samples). Statistical significance was assessed using Dunnett’s test: *P<0.05.

First, RESV-SULF was detected in HepG2 hepatocytes as previously reported [43], but not in the other two cell lines (Fig. 2A). Next, we could not detect DH-RESV and its metabolites in our tested cell cultures (data not shown), suggesting that mammalian cells cannot metabolize RESV into DH-RESV.

In contrast to RESV-SULF, we detected trans-RESV-3-O-GLUC and cis-RESV-3-O-GLUC in all three cell lines (Fig. 2B and C). Peak areas of trans-RESV-3-O-GLUC and cis-RESV-3-O-GLUC increased in a RESV dose-dependent manner in all three cell lines. These data indicate that human keratinocytes, human hepatocytes and mouse myoblasts metabolize RESV into trans-RESV-3-O-GLUC and cis-RESV-3-O-GLUC. Interestingly, peak area ratio of cis-RESV-3-O-GLUC/trans-RESV-3-O-GLUC is larger in HaCaT than in the other two cell types (HaCaT keratinocytes 11, while C2C12 myocytes and HepG2 hepatocytes 0.5 and 0.8, respectively when treated with 20 µM trans-RESV). The HaCaT cell line has a different metabolic profile regarding trans-RESV-3-O-GLUC and cis-RESV-3-O-GLUC compared with the other two lines. Mouse tissue and cell line data suggest that sulfation of RESV occurs mainly in liver and the glucuronidation of RESV can take place not only in liver, but also in skin.

### Resveratrol absorption efficiency through skin using different bases

**Figure 3:**
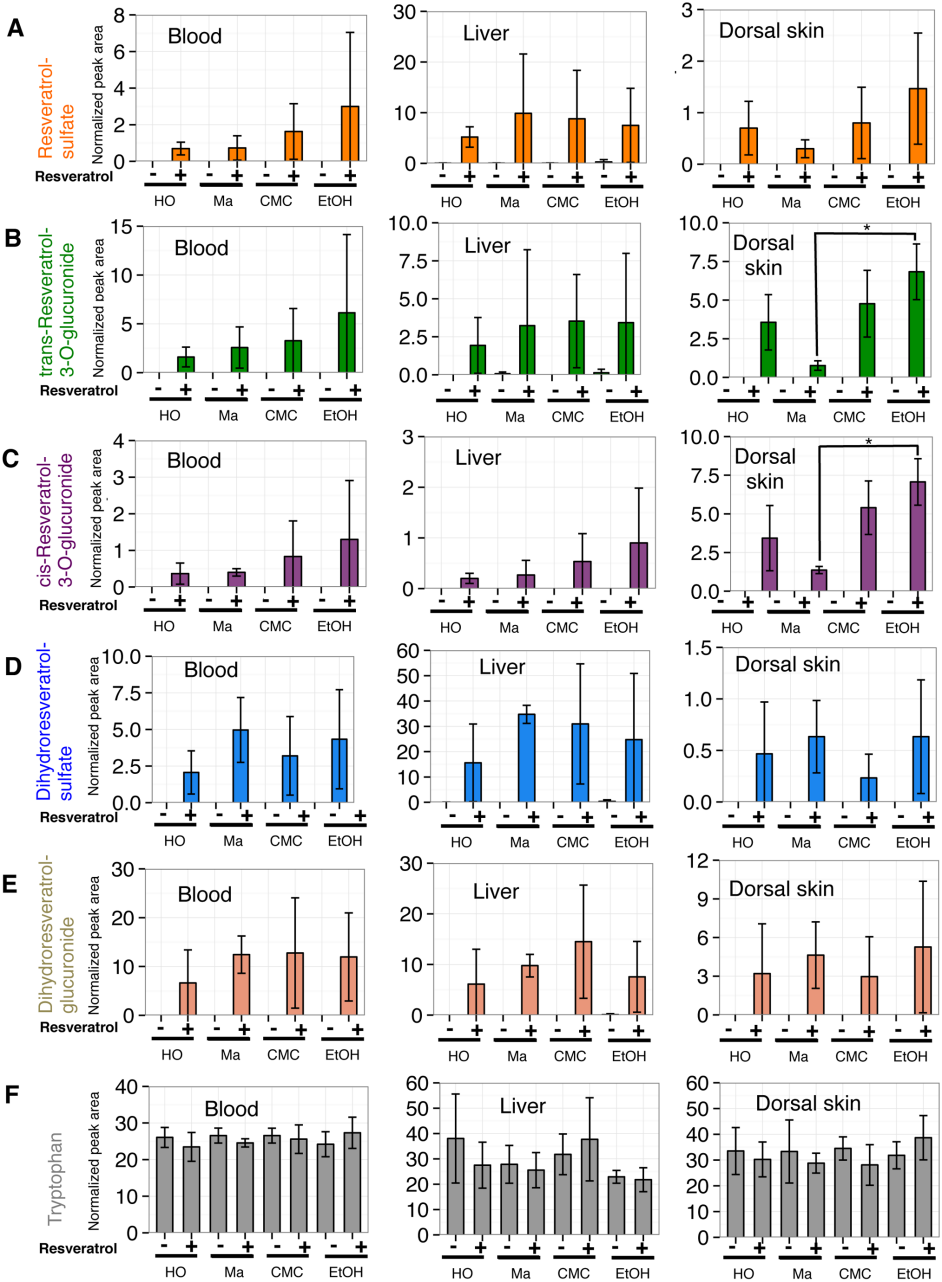
Absorption efficiency of RESV through mouse skin using 3 bases in different tissues. One mg of RESV dissolved in ethanol was applied directly (EtOH) or mixed with hydrophilic ointment (HO), macrogol (Ma) or CMC gel (CMC), and swabbed on mouse dorsal skin. After 4 h mice were sacrificed, metabolites were extracted from tissues and analyzed by LC-MS. Peak areas of RESV-SULF (**A**), trans-RESV-3-O-GLUC (**B**), cis-RESV-3-O-GLUC (**C**), DH-RESV-SULF (**D**), DH-RESV-GLUC (**E**) and tryptophan (**F**) were normalized using peak areas of spiked internal standards (HEPES and PIPES). Peak areas for each compound are presented as means ± SD of 3 mice. Statistical significance was assessed with Tukey’s test: *P<0.05.

Although we observed that RESV was absorbed through skin using ethanol as the solvent (Fig. 1), this would not be practical for clinical applications. We evaluated 3 different bases for efficiency of RESV skin absorption in mouse tissues. As RESV and its metabolites were barely detectable in muscle in our preliminary study, using bases (data not shown), we measured RESV metabolites in blood, liver and dorsal skin 4 h after transdermal treatment (Fig. 3). Bases used in this experiment were hydrophilic ointment, macrogol gel, and CMC gel. Hydrophilic ointment is a petrolatum-modified base. Macrogol is a mixture of polyethylene glycol 400 and 4000. CMC gel is mix of carboxymethylcellulose sodium, glycerol, and water. Macrogol and CMC gel are water-soluble bases. RESV dissolved in ethanol was mixed with these bases. Bases with ethanol alone were used as the control. Tryptophan levels of tissue samples are provided as a control (Fig. 3F).

First, using RESV dissolved in ethanol, RESV metabolites were robustly detected (Figs. 1 and 3). Samples treated with RESV mixed with each base showed similar, but distinct levels of RESV metabolites (Fig. 3).

In blood samples (Fig. 3, left column), the highest levels of RESV-SULF (Fig. 3A), trans-RESV-3-O-GLUC (Fig. 3B), and cis-RESV-3-O-GLUC (Fig. 3C) were detected in mice treated with RESV dissolved in ethanol. Blood samples after RESV treatment with hydrophilic ointment showed the smallest peaks for all RESV metabolites compared to the other 2 bases and RESV dissolved in ethanol. In liver samples (Fig. 3, middle column) RESV and DH-RESV metabolite peaks from RESV in hydrophilic ointment were also the smallest compared to other bases.

Samples treated with RESV in water-soluble bases (macrogol or CMC gel) displayed similar levels of DH-RESV-SULF and DH-RESV-GLUC in blood, and RESV-SULF, trans-RESV-3-O-GLUC, cis-RESV-3-O-GLUC, DH-RESV-SULF and DH-RESV-GLUC in liver, compared to those where RESV was dissolved in ethanol.

In dorsal skin samples (Fig. 3, right columns), RESV-SULF, trans-RESV-3-O-GLUC, and cis-RESV-3-O-GLUC were found most abundantly when RESV was dissolved in ethanol and less abundantly when RESV was mixed with macrogol. DH-RESV-SULF and DH-RESV-GLUC were detected most abundantly in RESV mixed with macrogol samples among the 3 tested bases.

Collectively, these results suggest that among the bases used in this study, water soluble bases, macrogol or CMC gel, exhibited efficiencies of transdermal RESV absorption and penetration, comparable to that of RESV dissolved in ethanol.

Using human blood metabolome analysis methods described previously [44], we evaluated RESV metabolites in blood from a couple of human volunteers after RESV oral or skin administration using different bases. RESV metabolites in human blood could be detected after oral, but not skin administration (data not shown).

### Effect of RESV after application to skin on ear edema model

**Figure 4:**
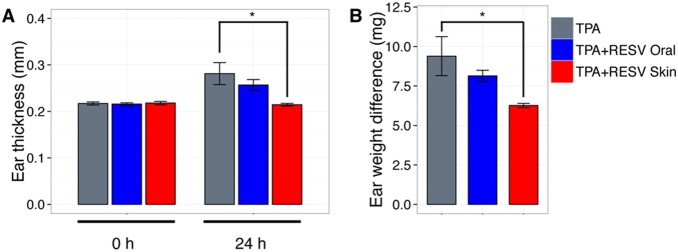
Anti-inflammatory effect of RESV on TPA-induced edema in mouse ear. To induce ear edema in mice, 1 µg TPA was topically applied on the inner and outer surface of the right ear. 30 min after TPA treatment, 1 mg of RESV was topically applied to the ear or administered orally. (**A**) Ear thickness was measured before (0 h) and after (24 h) application of TPA. (**B**) Edema was also measured as the difference between the weights of left (control) and right (TPA treated) ear punches 24 h after application of TPA. Results are expressed as the mean ± SD of 3–4 mice. Statistical differences were assessed using Tukey’s test: *P<0.05.

To evaluate beneficial effects of percutaneous administration of RESV, we used a mouse model of acute skin inflammation after TPA treatment. Anti-inflammatory activity of RESV after oral or skin administration in TPA-induced ear edema was measured as the difference in ear thickness and weight between the two groups. Before TPA treatment ear thickness of mice was 0.22 mm in each group (Fig. 4A). Ear thickness increased to 0.30 mm within 24 h after topical TPA treatment. RESV dermal application showed a significant decrease in ear thickness (0.21 mm) compared to the control group, as observed in a previous study [41]. In contrast, oral RESV administration did not show a significant change of ear thickness (0.26 mm). Ear weight was significantly lower in the transdermal RESV group (6.3 mg) compared to TPA-treated mice (9.4 mg), whereas oral RESV treatment had less effect (8.1 mg) (Fig. 4B). These data suggest that skin treatment with RESV inhibited TPA-induced ear edema, but that oral RESV treatment did not.

## Discussion

We analyzed the metabolism of RESV after oral or dermal administration. Metabolomics is a recently developed tool to detect and semi-quantify small metabolites [45], [46]. Indeed, our metabolome data in mice include various RESV metabolites, such as RESV-SULF, trans-RESV-3-O-GLUC, cis-RESV-3-O-GLUC, DH-RESV, etc., some of which have been previously described in mouse and mammalian studies [31], [47], [48].

We determined that RESV can be efficiently absorbed through skin in mice. One of the issues regarding human treatment with RESV is effective delivery of RESV. Numerous studies have examined oral administration in mice [49], rats [50], pigs [47], and humans [25]. Little is known about metabolism of transdermally-absorbed RESV, although some reports have described the existence of glucuronosyltransferases (UGTs) in the skin [51], dermal absorption of RESV *in vitro* [32], [52], and its antioxidant activity in mouse [53], [54] and human skin [55].

In this study we analyzed and compared the tissue distribution of RESV metabolites between oral and dermal administration. Using this approach, we identified hydrophilic bases that may be suitable for efficient transdermal absorption of RESV in mice, with possible future clinical applications. Since skin permeability of chemicals is different between humans and mice [56], absorption efficiency of RESV in human skin is not clear. Expression patterns of transporter proteins and kidney excretion of xenobiotics are also different between them [57]. Also in our small human trial we could not detect RESV and its metabolites in blood after dermal application. These results illustrate the need to explore human applications.

**Figure 5:**
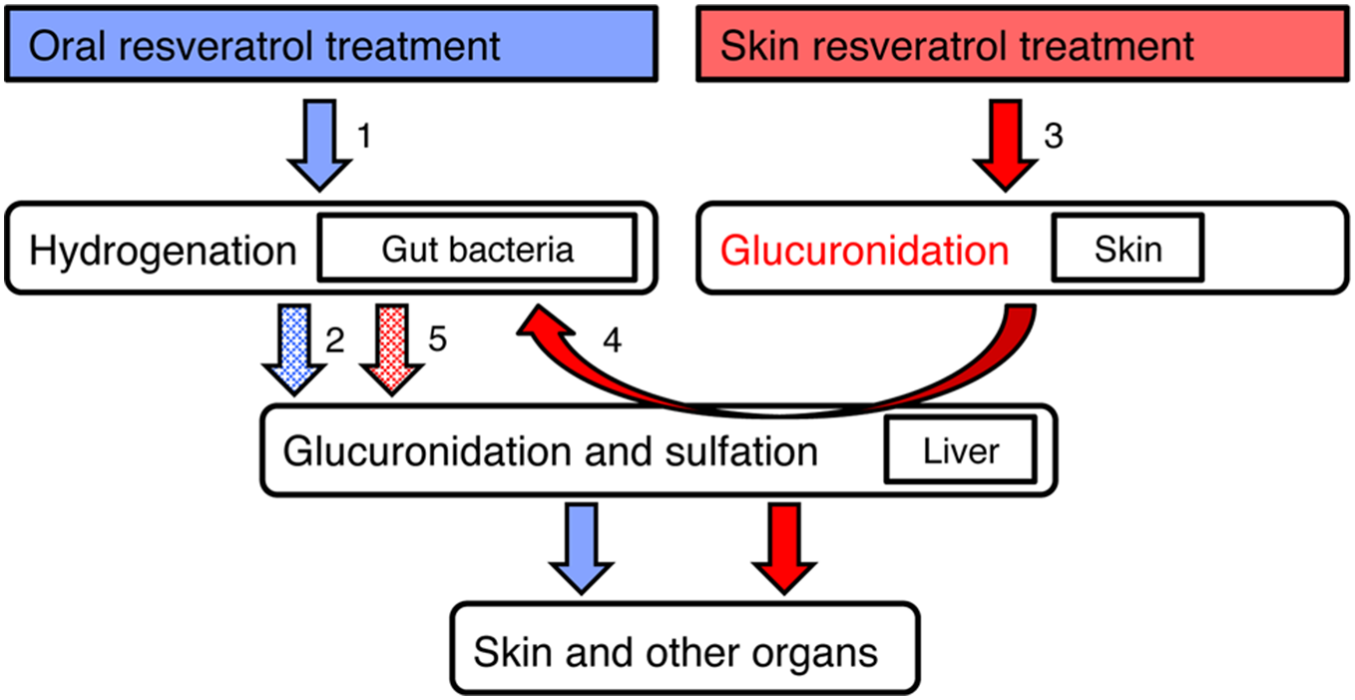
Model of RESV metabolism after oral and transdermal administration. Arrows indicate metabolism of RESV and dashed arrows indicate predicted metabolism of DH-RESV. Numbers beside the arrows are explained in the text.

Unexpectedly, we noticed that distribution profiles of RESV-SULF, trans-RESV-3-O-GLUC, cis-RESV-3-O-GLUC, and even DH-RESV after dermal application are quite similar to those observed after oral administration. Our metabolomic approach clearly detected DH-RESV metabolites in various tissues of mice, but not in three cell culture lines tested. These data imply that dermally absorbed RESV might also be metabolized into DH-RESV by gut bacteria (Fig. 5). After oral RESV administration, part of RESV is absorbed and metabolized by the liver into sulfated and glucuronidated forms. Unabsorbed RESV can be metabolized by gut bacteria into DH-RESV (Fig. 5 arrow 1), which then can be absorbed and transformed into DH-RESV-SULF and DH-RESV-GLUC by the liver (Fig. 5 dashed arrow 2). Enterohepatic circulation can enhance levels of RESV, DH-RESV and their metabolites. After topical application of RESV, RESV is absorbed and transformed into RESV glucuronides by skin cells (Fig. 5 arrow 3), followed by entry into the circulatory system. In liver, RESV can be modified into RESV-SULF and RESV glucuronides. Once excreted into the gut, the double bond in RESV and its metabolites can be reduced by gut bacteria, resulting in DH-RESV and its metabolites (Fig. 5 arrow 4). Enterohepatic circulation should be crucial for the absorption of DH-RESV and its metabolites into the body (Fig. 5 arrow 5).

Our comparison of oral and dermal administration of RESV *in vivo* identified distinct profiles of RESV metabolite distribution; the glucuronidation of RESV in skin after dermal application, but not its sulfation. These data are consistent with our observations *in vitro*. RESV glucuronides, but not RESV-SULF, are clearly detected in cultured skin cells, while both forms are found in cultured liver cells. These findings suggest that skin itself metabolizes RESV into the glucuronidated, but not the sulfated forms. Sulfation and glucuronidation of RESV are catalyzed by sulfotransferase SULT1 [58] and UDP-glucuronosyltransferases [59], respectively. It is also well known that RESV-SULF is generated by various cell types, not only liver, but also in breast [60] and brain cancer cells [61], while data concerning cell types producing RESV glucuronides are still scarce [62]. This is the first report of RESV glucuronide formation by skin cells.

While rapid absorption and metabolism of RESV after oral dosing was previously reported [63], permeability and metabolism of RESV through skin were largely unknown. In addition, topical application of RESV on mouse ears actually inhibited ear edema, whereas oral administration showed a smaller effect. This result suggests that skin application of RESV is effective to prevent acute inflammation in skin.

Glucuronidation of xenobiotic molecules is generally thought to serve in detoxification [64]. On the other hand, mammalian tissues contain glucuronidases that hydrolyze glucuronic acid from substrates [65]. Tissue glucuronidases might regenerate intact RESV from RESV glucuronides. In this way, RESV glucuronides might serve as a RESV reserve in the body. A similar situation might be expected in the case of RESV sulfates, which could be hydrolyzed to RESV by tissue sulfatases [30].

In conclusion, our comparative analysis *in vivo* and *in vitro* between oral and skin administration will be valuable for future clinical applications of RESV.

## Supplementary Material

Raw LC-MS data in mzML format, as well as data regarding detected metabolites were submitted to the MetaboLights [66] repository (http://www.ebi.ac.uk/metabolights) under accession numbers MTBLS125 (Fig. 1), MTBLS127 (Fig. 2) and MTBLS126 (Fig. 3).

## Supporting Information

S1 Figure **Detection and identification of RESV and RESV-SULF by LC-MS.** Chromatograms are shown for 227.071 *m/z* (RESV) and 307.027 *m/z* (RESV-SULF) in (**A**) trans-Resveratrol, (**B**) trans-Resveratrol-3-O-sulfate standard compounds, and (**C**) mouse liver sample. MS/MS fragmentation patterns are similar, as reported previously (S1 Table); possible chemical structures are shown. In mouse and cell tissue samples we detect single, early eluting peaks for the 227.071 *m/z* (RESV) and 307.027 *m/z* (RESV-SULF). Since we cannot exclude the possibility that cis- and trans- forms, as well as resveratrol-sulfates sulfated at different positions are coeluting, we designate those peaks as resveratrol (RESV) and resveratrol-sulfate (RESV-SULF). MS/MS fragmentation patterns of mouse liver samples match that of trans-Resveratrol-3-O-sulfate, the major fragment being 227.071 *m/z* (RESV). As shown in (A) and (B), retention times of trans-Resveratrol and trans-Resveratrol-3-O-sulfate are almost identical. Furthermore, injection of 0.5 pmol of trans-Resveratrol or trans-Resveratrol-3-O-sulfate results in similar signal intensity for the 227.071 *m/z* ion. In case of trans-Resveratrol-3-O-sulfate injection, 227.071 *m/z* ion is detected at much lower intensity than the 307.027 *m/z* ion. This complicates quantification of small resveratrol amounts, which could be present in the samples. RESV and RESV-SULF peak areas correlate, except for dorsal skin where RESV was applied (S6 A Figure).
(TIF)

S2 Figure **Detection and identification of DH-RESV and DH-RESV-SULF by LC-MS.** Chromatograms are shown for the 229.087 *m/z* (DH-RESV) and 309.044 *m/z* (DH-RESV-SULF) peaks in (**A**) dihydroresveratrol standard compound and (**B**) mouse liver sample. The MS/MS fragmentation pattern of DH-RESV-SULF is similar to that reported previously (S1 Table). Possible chemical structures are shown. In mouse samples we detect a single, early eluting peak for at 309.044 *m/z* (DH-RESV-SULF). Since we cannot exclude the possibility that DH-RESV sulfates, sulfated at different positions, are coeluting, thus we designate the peak as dihydroresveratrol-sulfate (DH-RESV-SULF). Similarly to spectra for RESV and RESV-SULF (S1 Figure), the MS/MS fragmentation pattern of 309.044 *m/z* peak (DH-RESV-SULF) in mouse liver shows the major fragment at 229.087 *m/z* (DH-RESV). As shown in (A) and (B), retention times of DH-RESV and DH-RESV-SULF are almost identical. Furthermore, as in RESV and RESV-SULF (S1 Figure), the 229.087 *m/z* ion is detected at much lower intensity than the 309.044 *m/z* ion. This complicates quantification of traces of DH-RESV that could be present in the samples. DH-RESV and DH-RESV-SULF peak areas correlate in all tissues (S6 B Figure).
(TIF)

S3 Figure **Identification of four RESV glucuronides by LC-MS.** Chromatograms for the 403.104*m/z* (RESV glucuronide) and 218.103*m/z* (pantothenate) ions are shown in cis-RESV-3-O-GLUC (**A**), cis-RESV-4-O-GLUC (**B**), trans-RESV-3-O-GLUC (**C**), trans-RESV-4-O-GLUC (**D**) standard compounds, and mouse blood sample (**E**). Retention times for each resveratrol-glucuronide standard were compared with those of pantothenate to identify RESV glucuronide peaks detected in mouse and cell culture metabolome samples. The first resveratrol-glucuronide peak in mouse blood metabolome samples elutes just before the pantothenate peak, as in (**A**); thus, it was identified as cis-RESV-3-O-GLUC. The second peak elutes about 1.4min after the pantothenate peak in the mouse blood sample, as in (**C**); thus it was identified as trans-RESV-3-O-GLUC.
(TIF)

S4 Figure **Peak identification of RESV metabolites by LC-MS.** Chromatograms are shown for (**A**) 483.061*m/z* (RESV-SULF-GLUC), (**B**) 386.985*m/z* (RESV-DISULF) and (**C**) 579.135*m/z* (RESV-DIGLUC) in RESV-treated metabolome samples. RESV-SULF-GLUC elutes in multiple peaks (483.061 *m/z*) around 12–16 min. The MS/MS fragmentation pattern contains masses of RESV (227.071 *m/z*), RESV-SULF (307.027 *m/z*), and RESV-GLUC (403.103 *m/z*). For the RESV-DISULF and RESV-DIGLUC peaks, no MS/MS data could be obtained; thus these peaks were identified by generating chemical formulae using MZmine module. None of the RESV metabolite peaks were detected in untreated mouse samples (data not shown).
(TIF)

S5 Figure **Peak identification of DH-RESV metabolites by LC-MS.** Chromatograms are shown for (**A**) 405.121*m/z* (DH-RESV-GLUC) and (**B**) 485.076*m/z* (DH-RESV-SULF-GLUC) in RESV-treated metabolome samples. DH-RESV-GLUC elutes in single peak at about 6.6min. The MS/MS fragmentation pattern contains masses of DH-RESV (229.087 *m/z*) and glucuronide fragments (175.025 *m/z* and 113.024 *m/z*). Possible chemical structures are shown. For the two DH-RESV-SULF-GLUC peaks, no MS/MS data could be obtained; thus these peaks were identified by generating chemical formulae using MZmine module and from retention times similar to that of RESV-SULF-GLUC (S4 A Figure).
(TIF)

S6 Figure **Comparison of the RESV and DH-RESV normalized peak areas with their sulfated counterparts.** (**A**) RESV and RESV-SULF normalized peak areas correlate in RESV-treated mouse tissues, except for dorsal skin, where RESV was applied directly. This is most likely due to the RESV-SULF fragmentation pattern (S1 Figure). (**B**) DH-RESV and DH-RESV-SULF normalized peak areas correlate in RESV-treated mouse tissues. This is most likely due to the RESV-SULF fragmentation pattern (S2 Figure).
(TIF)

S1 Table **RESV metabolites detected in the present study.**
(DOCX)

S2 Table **Methods and parameters used for data processing with MZmine 2.10.**
(DOCX)

## References

[1] Scully T (2012) Demography: To the limit. Nature 492:S2–3. 23222671 10.1038/492S2a

[2] Ray NF, Chan JK, Thamer M, Melton LJ 3rd (1997) Medical expenditures for the treatment of osteoporotic fractures in the United States in 1995: report from the National Osteoporosis Foundation. J Bone Miner Res 12:24–35. 9240722 10.1359/jbmr.1997.12.1.24

[3] Sasaki H (2008) Single pathogenesis of geriatric syndrome. Geriatr Gerontol Int 8:1–4. 18713182 10.1111/j.1447-0594.2008.00439.x

[4] Grinberg LT, Thal DR (2010) Vascular pathology in the aged human brain. Acta Neuropathol 119:277–290. 20155424 10.1007/s00401-010-0652-7 PMC2831184

[5] Vanitallie TB (2003) Frailty in the elderly: contributions of sarcopenia and visceral protein depletion. Metabolism 52:22–26. 14577059 10.1016/s0026-0495(03)00297-x

[6] Harman D (1956) Aging: a theory based on free radical and radiation chemistry. J Gerontol 11:298–300. 13332224 10.1093/geronj/11.3.298

[7] Hayflick L, Moorhead PS (1961) The serial cultivation of human diploid cell strains. Exp Cell Res 25:585–621. 13905658 10.1016/0014-4827(61)90192-6

[8] Martin GM (2011) The biology of aging: 1985–2010 and beyond. FASEB J 25:3756–3762. 22046003 10.1096/fj.11-1102.ufm PMC4050331

[9] McCay CM, Crowell MF, Maynard LA (1935) The Effect of Retarded Growth Upon the Length of Life Span and Upon the Ultimate Body Size. J Nutr 10:63–79. 2520283

[10] Everitt AV, Rattan SIS, G**.** **LCD, R. dC** (2010) Calorie Restriction, Aging and Longevity. Dordrecht, The Netherlands: Springer.

[11] Lin SJ, Ford E, Haigis M, Liszt G, Guarente L (2004) Calorie restriction extends yeast life span by lowering the level of NADH. Genes Dev 18:12–16. 14724176 10.1101/gad.1164804 PMC314267

[12] Imai S, Armstrong CM, Kaeberlein M, Guarente L (2000) Transcriptional silencing and longevity protein Sir2 is an NAD-dependent histone deacetylase. Nature 403:795–800. 10693811 10.1038/35001622

[13] Nerurkar PV, Nerurkar VR (2008) Respected Sir(2): magic target for diabetes. Cellscience 4:82–96. 20577646 PMC2890243

[14] Anderson RM, Bitterman KJ, Wood JG, Medvedik O, Sinclair DA (2003) Nicotinamide and PNC1 govern lifespan extension by calorie restriction in Saccharomyces cerevisiae. Nature 423:181–185. 12736687 10.1038/nature01578 PMC4802858

[15] Hardie DG, Ross FA, Hawley SA (2012) AMPK: a nutrient and energy sensor that maintains energy homeostasis. Nat Rev Mol Cell Biol 13:251–262. 22436748 10.1038/nrm3311 PMC5726489

[16] Dirks AJ, Leeuwenburgh C (2006) Caloric restriction in humans: potential pitfalls and health concerns. Mech Ageing Dev 127:1–7. 16226298 10.1016/j.mad.2005.09.001

[17] Nagai M, Kuriyama S, Kakizaki M, Ohmori-Matsuda K, Sugawara Y, et al (2010) Effect of Age on the Association between Body Mass Index and All-Cause Mortality: The Ohsaki Cohort Study. Journal of Epidemiology 20:398–407. 20699601 10.2188/jea.JE20090204 PMC3900835

[18] Martin-Montalvo A, Mercken EM, Mitchell SJ, Palacios HH, Mote PL, et al (2013) Metformin improves healthspan and lifespan in mice. Nat Commun 4:2192. 23900241 10.1038/ncomms3192 PMC3736576

[19] Price NL, Gomes AP, Ling AJ, Duarte FV, Martin-Montalvo A, et al (2012) SIRT1 is required for AMPK activation and the beneficial effects of resveratrol on mitochondrial function. Cell Metab 15:675–690. 22560220 10.1016/j.cmet.2012.04.003 PMC3545644

[20] Mizutani K, Ikeda K, Kawai Y, Yamori Y (2000) Resveratrol attenuates ovariectomy-induced hypertension and bone loss in stroke-prone spontaneously hypertensive rats. J Nutr Sci Vitaminol (Tokyo) 46:78–83. 10885794 10.3177/jnsv.46.78

[21] Vang O, Ahmad N, Baile CA, Baur JA, Brown K, et al (2011) What is new for an old molecule? Systematic review and recommendations on the use of resveratrol. PLoS One 6:e19881. 21698226 10.1371/journal.pone.0019881 PMC3116821

[22] Lagouge M, Argmann C, Gerhart-Hines Z, Meziane H, Lerin C, et al (2006) Resveratrol improves mitochondrial function and protects against metabolic disease by activating SIRT1 and PGC-1alpha. Cell 127:1109–1122. 17112576 10.1016/j.cell.2006.11.013

[23] Jang M, Cai L, Udeani GO, Slowing KV, Thomas CF, et al (1997) Cancer chemopreventive activity of resveratrol, a natural product derived from grapes. Science 275:218–220. 8985016 10.1126/science.275.5297.218

[24] Baur JA, Sinclair DA (2006) Therapeutic potential of resveratrol: the in vivo evidence. Nat Rev Drug Discov 5:493–506. 16732220 10.1038/nrd2060

[25] Timmers S, Auwerx J, Schrauwen P (2012) The journey of resveratrol from yeast to human. Aging (Albany NY) 4:146–158. 22436213 10.18632/aging.100445 PMC3348475

[26] Vang O (2013) What is new for resveratrol? Is a new set of recommendations necessary? Ann N Y Acad Sci 1290:1–11. 23855460 10.1111/nyas.12173

[27] Maier-Salamon A, Hagenauer B, Reznicek G, Szekeres T, Thalhammer T, et al (2008) Metabolism and disposition of resveratrol in the isolated perfused rat liver: role of Mrp2 in the biliary excretion of glucuronides. J Pharm Sci 97:1615–1628. 17724663 10.1002/jps.21057

[28] Jung CM, Heinze TM, Schnackenberg LK, Mullis LB, Elkins SA, et al (2009) Interaction of dietary resveratrol with animal-associated bacteria. FEMS Microbiol Lett 297:266–273. 19566680 10.1111/j.1574-6968.2009.01691.x

[29] Maier-Salamon A, BÖHmdorfer M, Thalhammer T, Szekeres T, Jaeger W (2011) Hepatic Glucuronidation of Resveratrol: Interspecies Comparison of Enzyme Kinetic Profiles in Human, Mouse, Rat, and Dog. Drug Metabolism and Pharmacokinetics 26:364–373. 21512262 10.2133/dmpk.dmpk-11-rg-006

[30] Maier-Salamon A, Bohmdorfer M, Riha J, Thalhammer T, Szekeres T, et al (2013) Interplay between metabolism and transport of resveratrol. Ann N Y Acad Sci 1290:98–106. 23855471 10.1111/nyas.12198

[31] Bode LM, Bunzel D, Huch M, Cho GS, Ruhland D, et al (2013) In vivo and in vitro metabolism of trans-resveratrol by human gut microbiota. Am J Clin Nutr 97:295–309. 23283496 10.3945/ajcn.112.049379

[32] Hung CF, Lin YK, Huang ZR, Fang JY (2008) Delivery of resveratrol, a red wine polyphenol, from solutions and hydrogels via the skin. Biol Pharm Bull 31:955–962. 18451526 10.1248/bpb.31.955

[33] Ministry of Health LaW (2011) The Japanese Pharmacopoeia 16th edition.

[34] Boocock DJ, Faust GE, Patel KR, Schinas AM, Brown VA, et al (2007) Phase I dose escalation pharmacokinetic study in healthy volunteers of resveratrol, a potential cancer chemopreventive agent. Cancer Epidemiol Biomarkers Prev 16:1246–1252. 17548692 10.1158/1055-9965.EPI-07-0022

[35] Knowles BB, Howe CC, Aden DP (1980) Human hepatocellular carcinoma cell lines secrete the major plasma proteins and hepatitis B surface antigen. Science 209:497–499. 6248960 10.1126/science.6248960

[36] Boukamp P, Petrussevska RT, Breitkreutz D, Hornung J, Markham A, et al (1988) Normal keratinization in a spontaneously immortalized aneuploid human keratinocyte cell line. J Cell Biol 106:761–771. 2450098 10.1083/jcb.106.3.761 PMC2115116

[37] Yaffe D, Saxel O (1977) Serial passaging and differentiation of myogenic cells isolated from dystrophic mouse muscle. Nature 270:725–727. 563524 10.1038/270725a0

[38] Pluskal T, Nakamura T, Villar-Briones A, Yanagida M (2010) Metabolic profiling of the fission yeast S. pombe: quantification of compounds under different temperatures and genetic perturbation. Mol Biosyst 6:182–198. 20024080 10.1039/b908784b

[39] Pluskal T, Castillo S, Villar-Briones A, Oresic M (2010) MZmine 2: modular framework for processing, visualizing, and analyzing mass spectrometry-based molecular profile data. BMC Bioinformatics 11:395. 20650010 10.1186/1471-2105-11-395 PMC2918584

[40] Pluskal T, Uehara T, Yanagida M (2012) Highly accurate chemical formula prediction tool utilizing high-resolution mass spectra, MS/MS fragmentation, heuristic rules, and isotope pattern matching. Anal Chem 84:4396–4403. 22497521 10.1021/ac3000418

[41] Bralley EE, Greenspan P, Hargrove JL, Wicker L, Hartle DK (2008) Topical anti-inflammatory activity of Polygonum cuspidatum extract in the TPA model of mouse ear inflammation. Journal of Inflammation 5:1. 18261214 10.1186/1476-9255-5-1 PMC2267461

[42] Maier-Salamon A, Bohmdorfer M, Thalhammer T, Szekeres T, Jaeger W (2011) Hepatic glucuronidation of resveratrol: interspecies comparison of enzyme kinetic profiles in human, mouse, rat, and dog. Drug Metab Pharmacokinet 26:364–373. 21512262 10.2133/dmpk.dmpk-11-rg-006

[43] Lancon A, Hanet N, Jannin B, Delmas D, Heydel JM, et al (2007) Resveratrol in human hepatoma HepG2 cells: metabolism and inducibility of detoxifying enzymes. Drug Metab Dispos 35:699–703. 17287390 10.1124/dmd.106.013664

[44] Chaleckis R, Ebe M, Pluskal T, Murakami I, Kondoh H, et al (2014) Unexpected similarities between the Schizosaccharomyces and human blood metabolomes, and novel human metabolites. Mol Biosyst 10:2538–2551. 25010571 10.1039/c4mb00346b

[45] Kuehnbaum NL, Britz-McKibbin P (2013) New advances in separation science for metabolomics: resolving chemical diversity in a post-genomic era. Chem Rev 113:2437–2468. 23506082 10.1021/cr300484s

[46] Pluskal T, Hayashi T, Saitoh S, Fujisawa A, Yanagida M (2011) Specific biomarkers for stochastic division patterns and starvation-induced quiescence under limited glucose levels in fission yeast. FEBS J 278:1299–1315. 21306563 10.1111/j.1742-4658.2011.08050.x PMC3123465

[47] Azorin-Ortuno M, Yanez-Gascon MJ, Vallejo F, Pallares FJ, Larrosa M, et al (2011) Metabolites and tissue distribution of resveratrol in the pig. Mol Nutr Food Res 55:1154–1168. 21710561 10.1002/mnfr.201100140

[48] Juan ME, Maijo M, Planas JM (2010) Quantification of trans-resveratrol and its metabolites in rat plasma and tissues by HPLC. J Pharm Biomed Anal 51:391–398. 19406597 10.1016/j.jpba.2009.03.026

[49] Vitrac X, Desmouliere A, Brouillaud B, Krisa S, Deffieux G, et al (2003) Distribution of [14C]-trans-resveratrol, a cancer chemopreventive polyphenol, in mouse tissues after oral administration. Life Sci 72:2219–2233. 12628442 10.1016/s0024-3205(03)00096-1

[50] Wenzel E, Soldo T, Erbersdobler H, Somoza V (2005) Bioactivity and metabolism of trans-resveratrol orally administered to Wistar rats. Mol Nutr Food Res 49:482–494. 15779067 10.1002/mnfr.200500003

[51] Sumida K, Kawana M, Kouno E, Itoh T, Takano S, et al (2013) Importance of UDP-glucuronosyltransferase 1A1 expression in skin and its induction by UVB in neonatal hyperbilirubinemia. Mol Pharmacol 84:679–686. 23950218 10.1124/mol.113.088112 PMC3807078

[52] Zillich OV, Schweiggert-Weisz U, Hasenkopf K, Eisner P, Kerscher M (2013) Release and in vitro skin permeation of polyphenols from cosmetic emulsions. Int J Cosmet Sci 35:491–501. 23763665 10.1111/ics.12072

[53] Reagan-Shaw S, Afaq F, Aziz MH, Ahmad N (2004) Modulations of critical cell cycle regulatory events during chemoprevention of ultraviolet B-mediated responses by resveratrol in SKH-1 hairless mouse skin. Oncogene 23:5151–5160. 15122319 10.1038/sj.onc.1207666

[54] Aziz MH, Afaq F, Ahmad N (2005) Prevention of ultraviolet-B radiation damage by resveratrol in mouse skin is mediated via modulation in survivin. Photochem Photobiol 81:25–31. 15469386 10.1562/2004-08-13-RA-274

[55] Wu Y, Jia LL, Zheng YN, Xu XG, Luo YJ, et al (2013) Resveratrate protects human skin from damage due to repetitive ultraviolet irradiation. J Eur Acad Dermatol Venereol 27:345–350. 22221158 10.1111/j.1468-3083.2011.04414.x

[56] Durrheim H, Flynn GL, Higuchi WI, Behl CR (1980) Permeation of hairless mouse skin I: Experimental methods and comparison with human epidermal permeation by alkanols. J Pharm Sci 69:781–786. 7391939 10.1002/jps.2600690709

[57] Chu X, Bleasby K, Evers R (2013) Species differences in drug transporters and implications for translating preclinical findings to humans. Expert Opin Drug Metab Toxicol 9:237–252. 23256482 10.1517/17425255.2013.741589

[58] Ung D, Nagar S (2009) Trans-resveratrol-mediated inhibition of beta-oestradiol conjugation in MCF-7 cells stably expressing human sulfotransferases SULT1A1 or SULT1E1, and human liver microsomes. Xenobiotica 39:72–79. 19219749 10.1080/00498250802604082

[59] Sabolovic N, Humbert AC, Radominska-Pandya A, Magdalou J (2006) Resveratrol is efficiently glucuronidated by UDP-glucuronosyltransferases in the human gastrointestinal tract and in Caco-2 cells. Biopharm Drug Dispos 27:181–189. 16477579 10.1002/bdd.498

[60] Miksits M, Wlcek K, Svoboda M, Thalhammer T, Ellinger I, et al (2010) Expression of sulfotransferases and sulfatases in human breast cancer: impact on resveratrol metabolism. Cancer Lett 289:237–245. 19747768 10.1016/j.canlet.2009.08.020

[61] Sun Z, Li H, Shu XH, Shi H, Chen XY, et al (2012) Distinct sulfonation activities in resveratrol-sensitive and resveratrol-insensitive human glioblastoma cells. FEBS J 279:2381–2392. 22540632 10.1111/j.1742-4658.2012.08617.x

[62] Wu B, Kulkarni K, Basu S, Zhang S, Hu M (2011) First-pass metabolism via UDP-glucuronosyltransferase: a barrier to oral bioavailability of phenolics. J Pharm Sci 100:3655–3681. 21484808 10.1002/jps.22568 PMC3409645

[63] Sale S, Verschoyle RD, Boocock D, Jones DJ, Wilsher N, et al (2004) Pharmacokinetics in mice and growth-inhibitory properties of the putative cancer chemopreventive agent resveratrol and the synthetic analogue trans 3,4,5,4′-tetramethoxystilbene. Br J Cancer 90:736–744. 14760392 10.1038/sj.bjc.6601568 PMC2409587

[64] de Santi C, Pietrabissa A, Mosca F, Pacifici GM (2000) Glucuronidation of resveratrol, a natural product present in grape and wine, in the human liver. Xenobiotica 30:1047–1054. 11197066 10.1080/00498250010002487

[65] Wawrzyniak CJ, Gallagher PM, D’Amore MA, Carter JE, Lund SD, et al (1989) DNA determinants of structural and regulatory variation within the murine beta-glucuronidase gene complex. Mol Cell Biol 9:4074–4078. 2779578 10.1128/mcb.9.9.4074-4078.1989 PMC362475

[66] Haug K, Salek RM, Conesa P, Hastings J, de Matos P, et al (2013) MetaboLights–an open-access general-purpose repository for metabolomics studies and associated meta-data. Nucleic Acids Res 41:D781–786. 23109552 10.1093/nar/gks1004 PMC3531110

