# Supplementary figures and images for: Metabolism of Skin-Absorbed Resveratrol into Its Glucuronized Form in Mouse Skin
Source: PLoS One. 2014 Dec 15;9(12):e115359. doi: 10.1371/journal.pone.0115359 (PMC4266648; doi:10.1371/journal.pone.0115359)

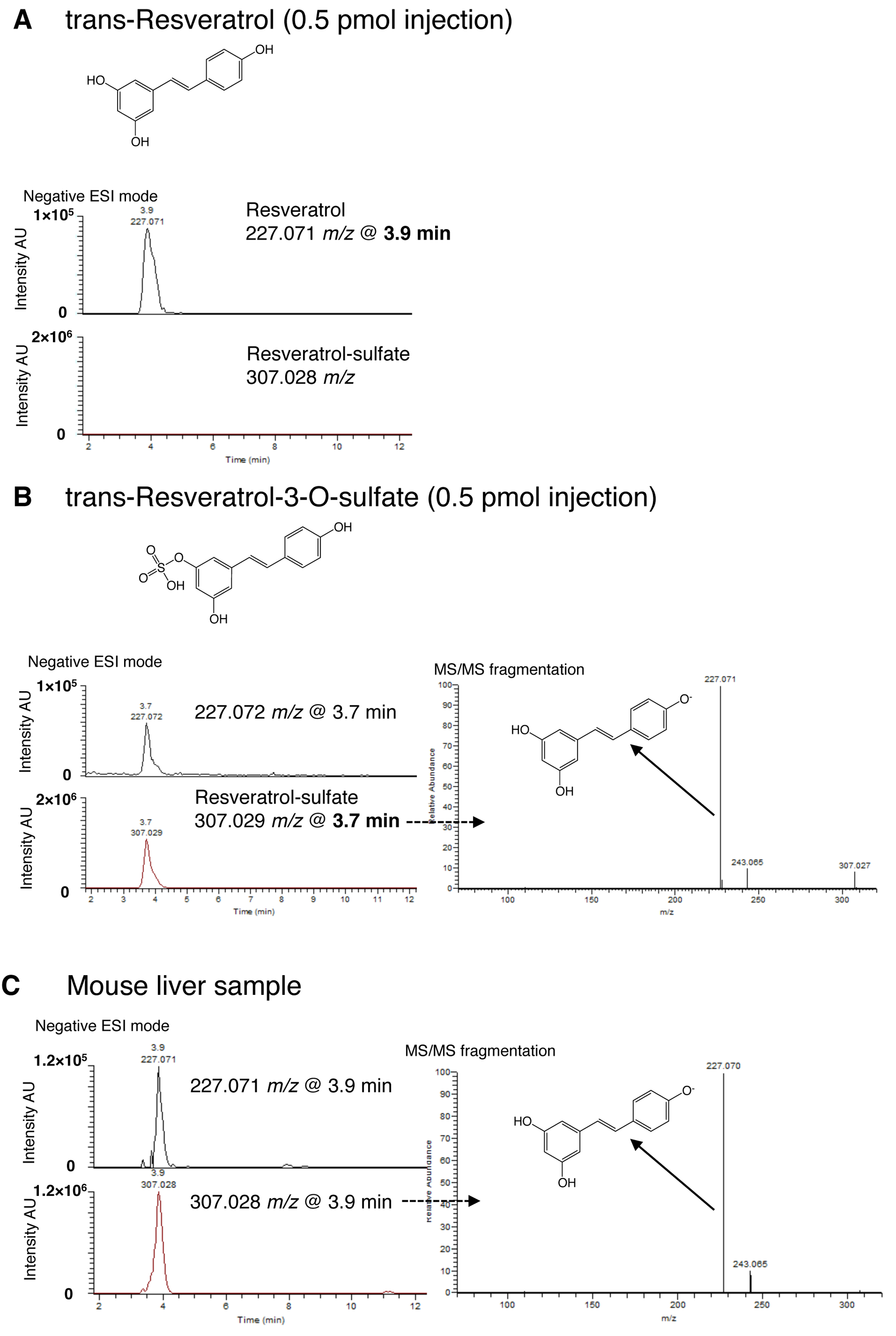

Supplement: S1 Figure — Detection and identification of RESV and RESV-SULF by LC-MS. Chromatograms are shown for 227.071 m/z (RESV) and 307.027 m/z (RESV-SULF) in (A) trans-Resveratrol, (B) trans-Resveratrol-3-O-sulfate standard compounds, and (C) mouse liver sample. MS/MS fragmentation patterns are similar, as reported previously (S1 Table); possible chemical structures are shown. In mouse and cell tissue samples we detect single, early eluting peaks for the 227.071 m/z (RESV) and 307.027 m/z (RESV-SULF). Since we cannot exclude the possibility that cis- and trans- forms, as well as resveratrol-sulfates sulfated at different positions are coeluting, we designate those peaks as resveratrol (RESV) and resveratrol-sulfate (RESV-SULF). MS/MS fragmentation patterns of mouse liver samples match that of trans-Resveratrol-3-O-sulfate, the major fragment being 227.071 m/z (RESV). As shown in (A) and (B), retention times of trans-Resveratrol and trans-Resveratrol-3-O-sulfate are almost identical. Furthermore, injection of 0.5 pmol of trans-Resveratrol or trans-Resveratrol-3-O-sulfate results in similar signal intensity for the 227.071 m/z ion. In case of trans-Resveratrol-3-O-sulfate injection, 227.071 m/z ion is detected at much lower intensity than the 307.027 m/z ion. This complicates quantification of small resveratrol amounts, which could be present in the samples. RESV and RESV-SULF peak areas correlate, except for dorsal skin where RESV was applied (S6 A Figure). (TIF) [file pone.0115359.s001.tif]

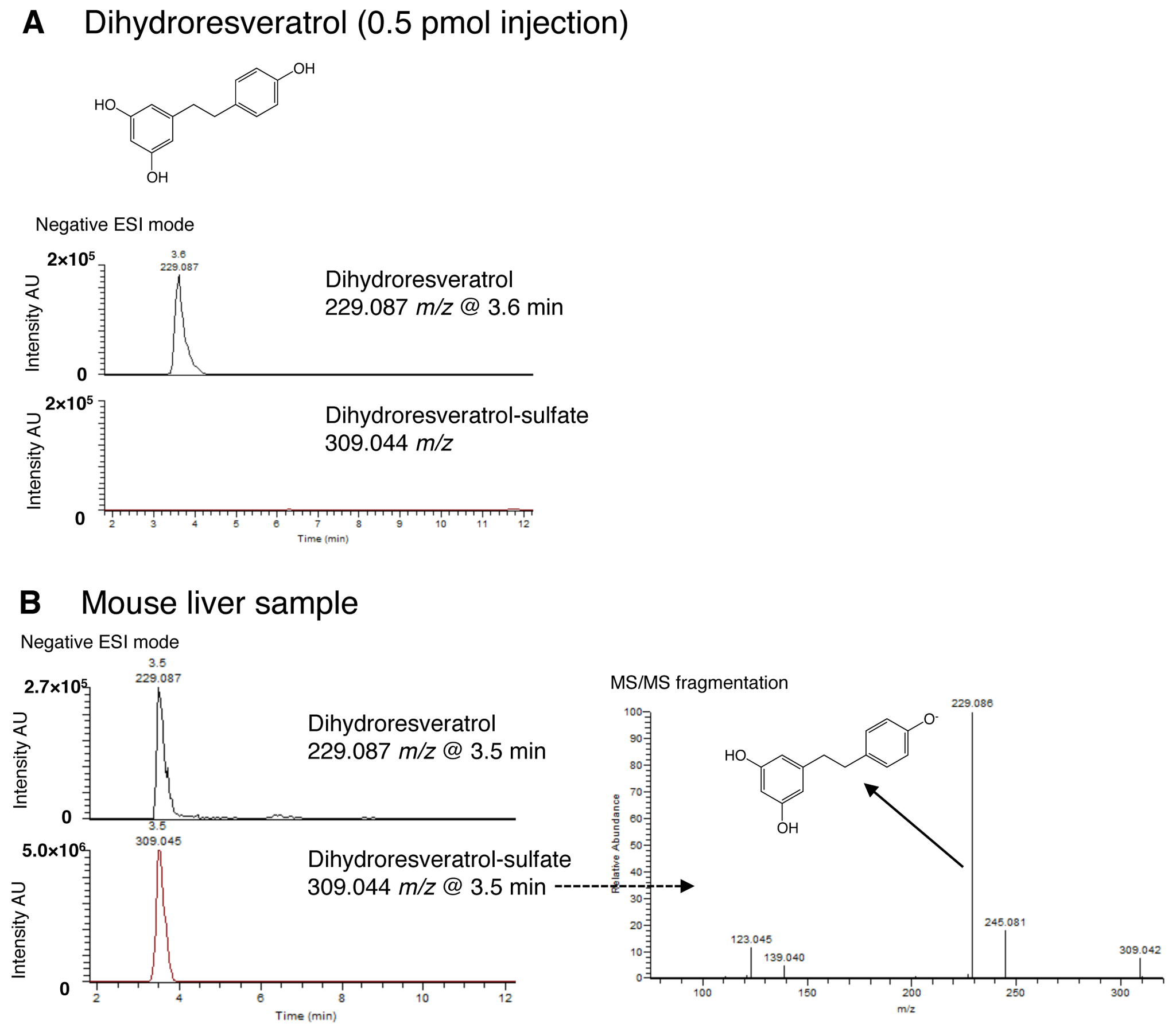

Supplement: S2 Figure — Detection and identification of DH-RESV and DH-RESV-SULF by LC-MS. Chromatograms are shown for the 229.087 m/z (DH-RESV) and 309.044 m/z (DH-RESV-SULF) peaks in (A) dihydroresveratrol standard compound and (B) mouse liver sample. The MS/MS fragmentation pattern of DH-RESV-SULF is similar to that reported previously (S1 Table). Possible chemical structures are shown. In mouse samples we detect a single, early eluting peak for at 309.044 m/z (DH-RESV-SULF). Since we cannot exclude the possibility that DH-RESV sulfates, sulfated at different positions, are coeluting, thus we designate the peak as dihydroresveratrol-sulfate (DH-RESV-SULF). Similarly to spectra for RESV and RESV-SULF (S1 Figure), the MS/MS fragmentation pattern of 309.044 m/z peak (DH-RESV-SULF) in mouse liver shows the major fragment at 229.087 m/z (DH-RESV). As shown in (A) and (B), retention times of DH-RESV and DH-RESV-SULF are almost identical. Furthermore, as in RESV and RESV-SULF (S1 Figure), the 229.087 m/z ion is detected at much lower intensity than the 309.044 m/z ion. This complicates quantification of traces of DH-RESV that could be present in the samples. DH-RESV and DH-RESV-SULF peak areas correlate in all tissues (S6 B Figure). (TIF) [file pone.0115359.s002.tif]

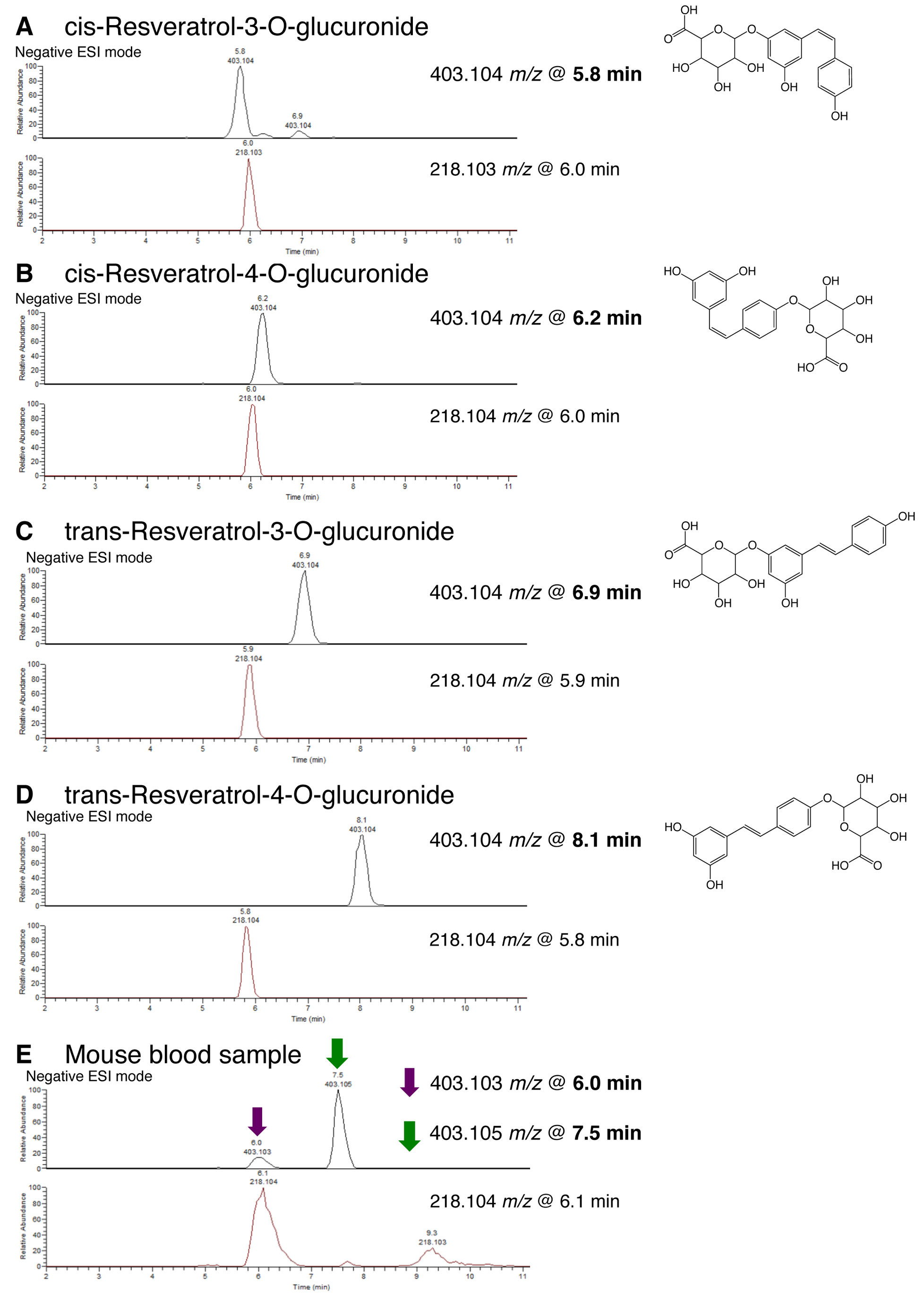

Supplement: S3 Figure — Identification of four RESV glucuronides by LC-MS. Chromatograms for the 403.104m/z (RESV glucuronide) and 218.103m/z (pantothenate) ions are shown in cis-RESV-3-O-GLUC (A), cis-RESV-4-O-GLUC (B), trans-RESV-3-O-GLUC (C), trans-RESV-4-O-GLUC (D) standard compounds, and mouse blood sample (E). Retention times for each resveratrol-glucuronide standard were compared with those of pantothenate to identify RESV glucuronide peaks detected in mouse and cell culture metabolome samples. The first resveratrol-glucuronide peak in mouse blood metabolome samples elutes just before the pantothenate peak, as in (A); thus, it was identified as cis-RESV-3-O-GLUC. The second peak elutes about 1.4min after the pantothenate peak in the mouse blood sample, as in (C); thus it was identified as trans-RESV-3-O-GLUC. (TIF) [file pone.0115359.s003.tif]

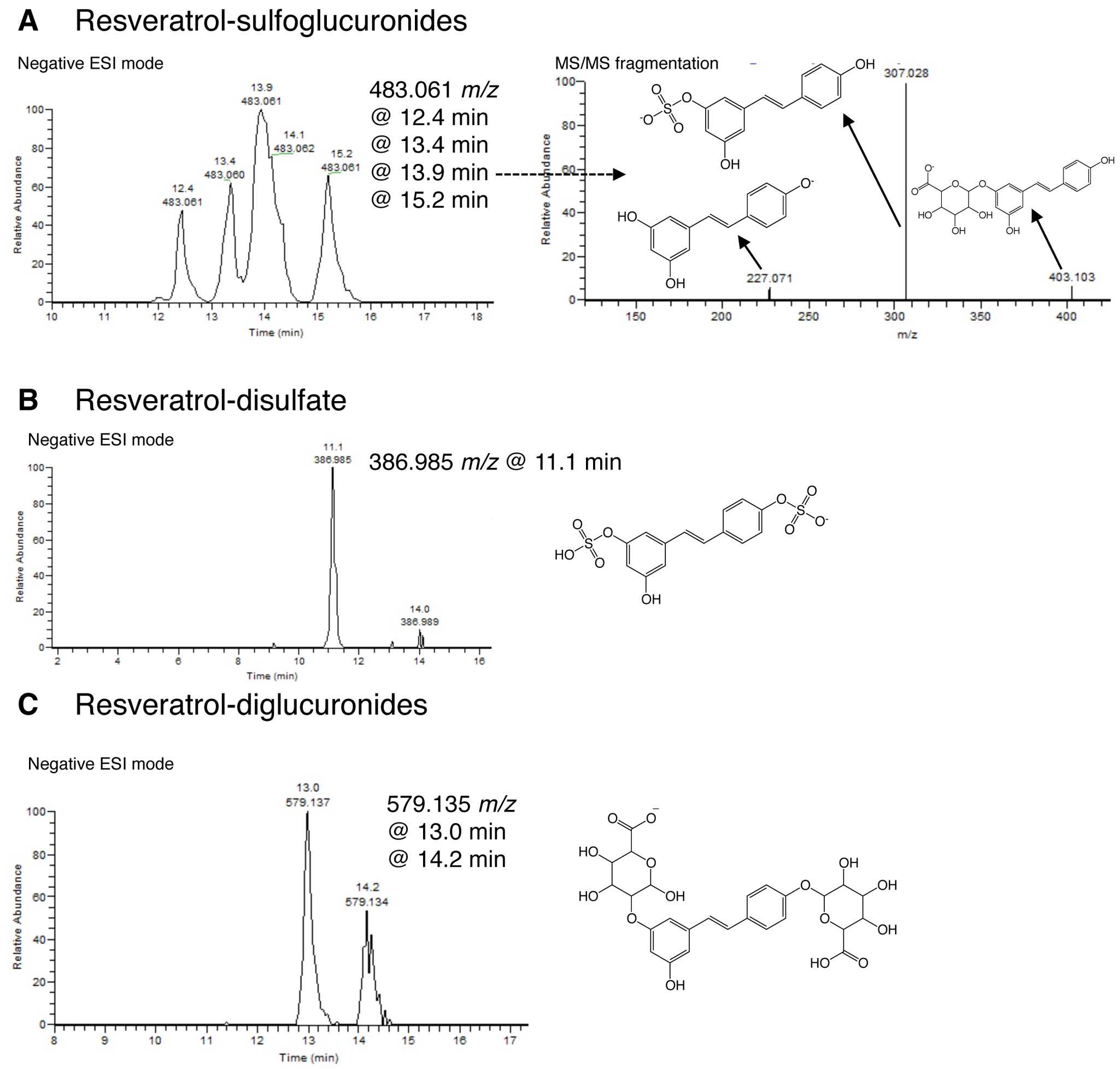

Supplement: S4 Figure — Peak identification of RESV metabolites by LC-MS. Chromatograms are shown for (A) 483.061m/z (RESV-SULF-GLUC), (B) 386.985m/z (RESV-DISULF) and (C) 579.135m/z (RESV-DIGLUC) in RESV-treated metabolome samples. RESV-SULF-GLUC elutes in multiple peaks (483.061 m/z) around 12–16 min. The MS/MS fragmentation pattern contains masses of RESV (227.071 m/z), RESV-SULF (307.027 m/z), and RESV-GLUC (403.103 m/z). For the RESV-DISULF and RESV-DIGLUC peaks, no MS/MS data could be obtained; thus these peaks were identified by generating chemical formulae using MZmine module. None of the RESV metabolite peaks were detected in untreated mouse samples (data not shown). (TIF) [file pone.0115359.s004.tif]

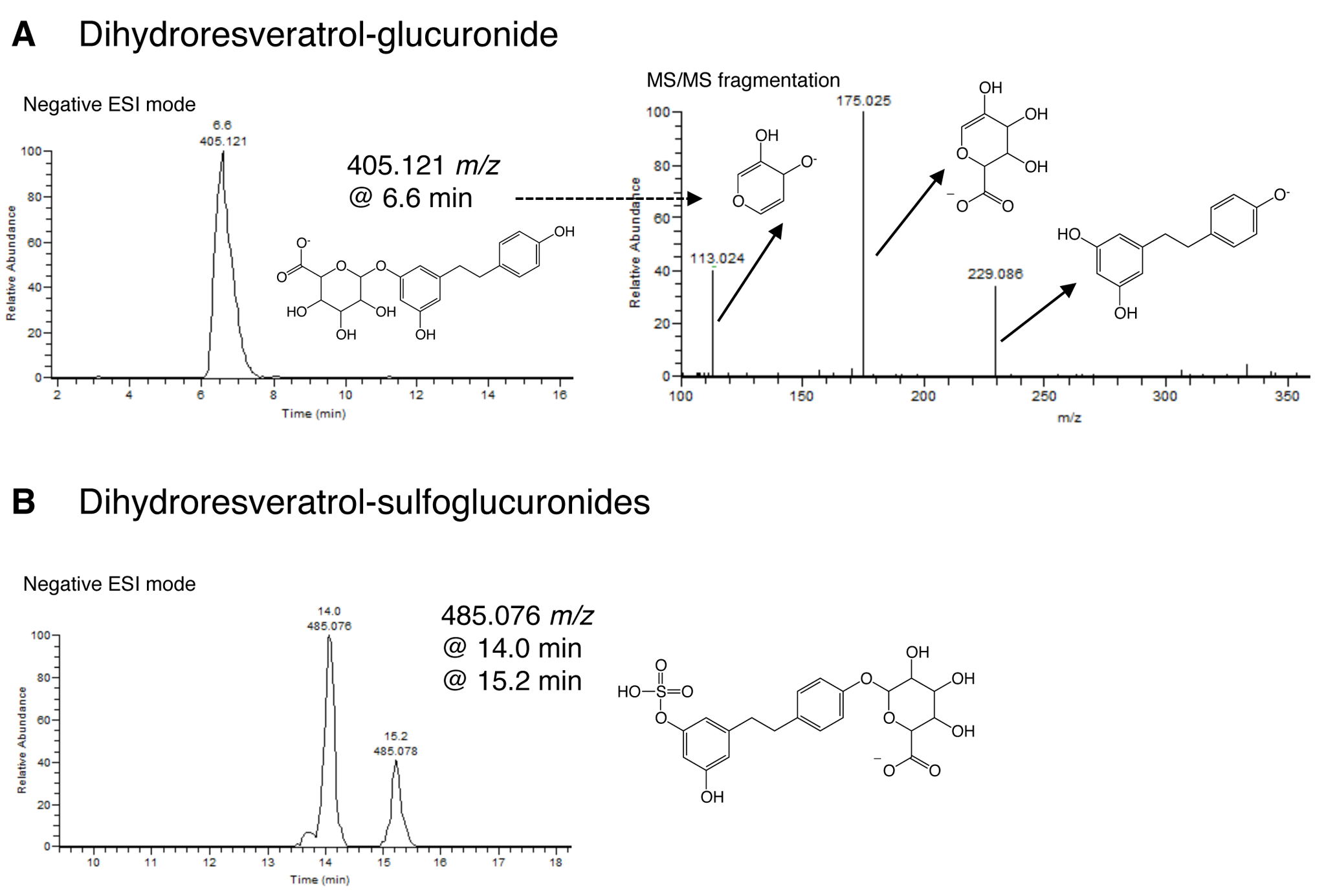

Supplement: S5 Figure — Peak identification of DH-RESV metabolites by LC-MS. Chromatograms are shown for (A) 405.121m/z (DH-RESV-GLUC) and (B) 485.076m/z (DH-RESV-SULF-GLUC) in RESV-treated metabolome samples. DH-RESV-GLUC elutes in single peak at about 6.6min. The MS/MS fragmentation pattern contains masses of DH-RESV (229.087 m/z) and glucuronide fragments (175.025 m/z and 113.024 m/z). Possible chemical structures are shown. For the two DH-RESV-SULF-GLUC peaks, no MS/MS data could be obtained; thus these peaks were identified by generating chemical formulae using MZmine module and from retention times similar to that of RESV-SULF-GLUC (S4 A Figure). (TIF) [file pone.0115359.s005.tif]

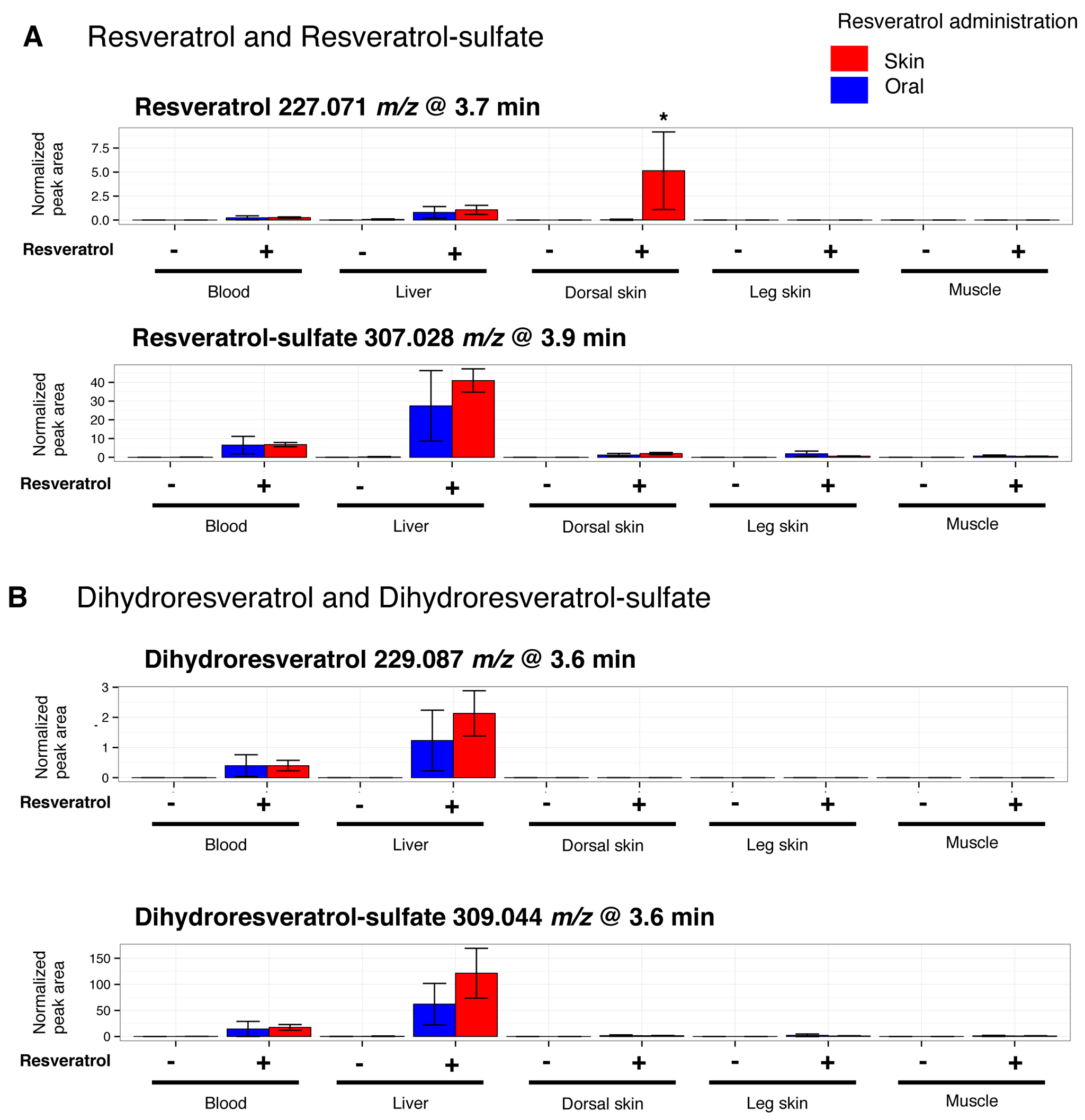

Supplement: S6 Figure — Comparison of the RESV and DH-RESV normalized peak areas with their sulfated counterparts. (A) RESV and RESV-SULF normalized peak areas correlate in RESV-treated mouse tissues, except for dorsal skin, where RESV was applied directly. This is most likely due to the RESV-SULF fragmentation pattern (S1 Figure). (B) DH-RESV and DH-RESV-SULF normalized peak areas correlate in RESV-treated mouse tissues. This is most likely due to the RESV-SULF fragmentation pattern (S2 Figure). (TIF) [file pone.0115359.s006.tif]
