# Supplementary material for: Metabolism of Skin-Absorbed Resveratrol into Its Glucuronized Form in Mouse Skin
Source: PLoS One. 2014 Dec 15;9(12):e115359. doi: 10.1371/journal.pone.0115359 (PMC4266648; doi:10.1371/journal.pone.0115359)
Supplement: S1 Table — RESV metabolites detected in the present study. (DOCX) [file pone.0115359.s007.docx]

**Table S1.** RESV metabolites detected in the present study.

| **Resveratrol metabolite** | **Formula** | **Negative ionization mode *m/z*, retention time (RT)** | **Reports and comments** |
| --- | --- | --- | --- |
| Resveratrol  (RESV) | C_14_H_12_O_3_ | 227.071 *m/z* RT 3.7 min | Quantification issue (Figure S1 and Figure S6) |
| Resveratrol-sulfate (RESV-SULF) | C_14_H_12_O_6_S | 307.028 *m/z* RT 3.9 min | Juan et al 2010 [1] (MS/MS data) |
| Dihydroresveratrol (DH-RESV) | C_14_H_14_O_3_ | 229.087 *m/z* RT 3.6 min | Quantification issue (Figure S2 and Figure S6) |
| Dihydroresveratrol-sulfate (DH-RESV-SULF) | C_14_H_14_O_6_S | 309.044 *m/z* RT 3.5 min | Wang et al 2005 [2] (MS/MS data) |
| cis-Resveratrol-3-O-glucuronide (cis-RESV-3-O-GLUC) | C_20_H_20_O_9_ | 403.104 *m/z* RT 6.0 min | Urpi-Sarda et al 2005 [3] (MS/MS data) |
| trans-Resveratrol-3-O-glucuronide (trans-RESV-3-O-GLUC) | C_20_H_20_O_9_ | 403.104 *m/z* RT 7.5 min | Urpi-Sarda et al 2005 [3] (MS/MS data) |
| Dihydroresveratrol-glucuronide (DH-RESV-GLUC) | C_20_H_22_O_9_ | 405.121 *m/z* RT 6.6 min | Azorin-Ortuno et al 2011 [4] (MS/MS data) |
| Resveratrol-sulfoglucuronide (RESV-SULF-GLUC) | C_20_H_20_O_12_S | 483.061 *m/z* RT 12 -16 min | Azorin-Ortuno et al 2011 [4] (MS/MS data) Detected in blood mouse blood samples |
| Dihydroresveratrol-sulfoglucuronide (DH-RESV-SULF-GLUC) | C_20_H_22_O_12_S | 485.076 *m/z* RT13 - 16 min | Detected in mouse liver samples |
| Resveratrol-disulfate (RESV-DISULF) | C_14_H_12_O_9_S_2_ | 386.985 *m/z* RT 11.1 min | Detected in mouse blood and liver samples |
| Resveratrol-diglucuronide (RESV-DIGLUC) | C_26_H_28_O_15_ | 579.136 *m/z* RT13 - 14 min | Detected in mouse skin samples |

1. Juan ME, Alfaras I, Planas JM (2010) Determination of dihydroresveratrol in rat plasma by HPLC. J Agric Food Chem 58: 7472-7475.

2. Wang D, Hang T, Wu C, Liu W (2005) Identification of the major metabolites of resveratrol in rat urine by HPLC-MS/MS. J Chromatogr B Analyt Technol Biomed Life Sci 829: 97-106.

3. Urpi-Sarda M, Jauregui O, Lamuela-Raventos RM, Jaeger W, Miksits M, et al. (2005) Uptake of diet resveratrol into the human low-density lipoprotein. Identification and quantification of resveratrol metabolites by liquid chromatography coupled with tandem mass spectrometry. Anal Chem 77: 3149-3155.

4. Azorin-Ortuno M, Yanez-Gascon MJ, Vallejo F, Pallares FJ, Larrosa M, et al. (2011) Metabolites and tissue distribution of resveratrol in the pig. Mol Nutr Food Res 55: 1154-1168.
