# Supplementary material for: Metabolism of Skin-Absorbed Resveratrol into Its Glucuronized Form in Mouse Skin
Source: PLoS One. 2014 Dec 15;9(12):e115359. doi: 10.1371/journal.pone.0115359 (PMC4266648; doi:10.1371/journal.pone.0115359)
Supplement: S2 Table — Methods and parameters used for data processing with MZmine 2.10. (DOCX) [file pone.0115359.s008.docx]

**Table S2.** Methods and parameters used for data processing with MZmine 2.10

| **Step** | **Parameter** | **Value** |
| --- | --- | --- |
| 1) Mass detection | | |
|  | Mass detection, mass detector Noise level (positive ionization mode) Noise level (negative ionization mode) | Exact mass 5E3 1E3 |
|  | Mass detection, MS level | 1 |
| 2) FTMS shoulder peaks filter | | |
|  | Mass resolution | 60 000 |
|  | Peak model function | Lorentzian extended |
| 3) Chromatogram builder | | |
|  | Min time span (min) | 0.1 |
|  | Min height | 1E4 |
|  | m/z tolerance | 0.003 m/z or 10 ppm |
| 4) Smoothing | | |
|  | Filter width | 9 |
| 5) Chromatogram deconvolution | | |
|  | Algorithm | Local minimum search |
|  | Chromatographic threshold | 70% |
|  | Search minimum in RT range (min) | 0.3 |
|  | Minimum relative height | 1 % |
|  | Minimum absolute height | 1E4 |
|  | Min ratio of peak top/edge | 1.5 |
|  | Peak duration range (min) | 0-20 |
| 6) Isotope peak grouper | | |
|  | m/z tolerance | 0.02 m/z or 20 ppm |
|  | Retention time tolerance | 0.1 min |
|  | Maximum charge | 2 |
|  | Representative isotope | Most intense |
| 7) Join aligner | | |
|  | m/z tolerance | 0.004 m/z or 10 ppm |
|  | Weight for m/z | 10 |
|  | Retention time tolerance | 0.5 min |
|  | Weight for RT | 10 |
| 8) Same RT and m/z range filling | | |
|  | m/z tolerance | 0.004 m/z or 10 ppm |
| 9) Custom database search | | |
| 10) Standard compound normalizer (HEPES, PIPES) | | |
|  | Normalization type | Weighted contribution of all standards |
|  | Peak measurement type | Peak area |
|  | m/z vs. RT balance | 10 |
